# A cassava protoplast system for screening genes associated with the response to *South African cassava mosaic virus*

**DOI:** 10.1186/s12985-020-01453-4

**Published:** 2020-11-23

**Authors:** Patience Chatukuta, Marie Emma Christine Rey

**Affiliations:** School of Molecular and Cell Biology, University of the Witwatersrand, Johannesburg, South Africa

**Keywords:** Cassava mosaic disease, Geminivirus, Protoplast, ubiquitin E3 ligase

## Abstract

**Background:**

The study of transient gene expression in cassava plants during virus infection using existing protocols is laborious and may take approximately fifteen weeks due to cassava’s recalcitrance to transformation. The combination of a protoplast system with CRISPR-mediated gene editing promises to shorten the turnaround time from plant tissue culture to high-throughput gene expression screening for candidate genes. Here, we detail a protocol for screening genes associated with the response to South African cassava mosaic virus (SACMV) in cassava protoplasts, with reference to the ubiquitin E3 ligase gene, *MeE3L*.

**Methods:**

Cassava protoplasts of model, and SACMV-susceptible and -tolerant genotypes, were transformed with SACMV infectious clones and/or a CRISPR-editing construct targeting the *MeE3L* using PEG4000-mediated transfection. DNA and RNA were extracted from transformed protoplasts at 24 h post-transfection. Relative SACMV DNA accumulation was determined via qPCR using *DpnI*-digested total DNA, *MeE3L* relative expression was determined via reverse transcriptase qPCR, and results were analysed using one-way ANOVA, Tukey’s HSD test and the 2^−ΔΔCT^statistical method. The *MeE3L *exonic region was sequenced on the ABI 3500XL Genetic Analyzer platform; and sequences were analysed for mutations using MAFTT and MEGA-X software. Construction of a phylogenetic tree was done using the Maximum Likelihood method and Jones-Taylor-Thornton (JTT) matrix-based model.

**Results:**

The differential expression of unedited and mutant *MeE3L* during SACMV infection of model, susceptible and tolerant cassava protoplasts was determined within 7 weeks after commencement of tissue culture. The study also revealed that SACMV DNA accumulation in cassava protoplasts is genotype-dependent and induces multiple mutations in the tolerant landrace *MeE3L* homolog. Notably, the susceptible cassava landrace encodes a RINGless *MeE3L*which is silenced by SACMV-induced mutations. SACMV also induces mutations which silence the *MeE3L* RING domain in protoplasts from and tolerant cassava landraces.

**Conclusions:**

This protocol presented here halves the turnaround time for high-throughput screening of genes associated with the host response to SACMV. It provides evidence that a cassava E3 ligase is associated with the response to SACMV and forms a basis for validation of these findings by *in planta *functional and interaction studies.

## Background

*South African cassava mosaic virus* is one of many cassava mosaic geminivirus species that affect the important food crop, cassava (*Manihot esculenta *Crantz), causing the distinct foliar symptoms characterised as cassava mosaic disease (CMD). CMD symptoms include mosaic pattern leaf chlorosis, curling, shape distortion and reduced size; which lead to production of few or no tubers [3, 66].

South African cassava mosaic virus (SACMV) occurrence was first reported in South Africa and Swaziland [11], then subsequently in Zimbabwe [15] and Madagascar [24]. This bipartite begomovirus with a single-stranded DNA genome was identified as a distinct geminivirus based on DNA A sequence comparison, serology, and amplification of a DNA B fragment; and it was shown to have high sequence similarity with tomato yellow leaf curl virus (TYLCV), a monopartite begomovirus [12, 13]. SACMV is transmitted by the whitefly species complex (*Bemisia tabaci *Genn.) [37] and perpetuated through infected stem cuttings used for propagation [21]. The effect of SACMV on cassava yield is dependent on the cassava genotype and environmental conditions, and it varies from no effect to total crop loss [66]. Major regional pandemics of CMD can cause major food security destabilisation, such as that which occurred in East and Central Africa in the 1990s [46, 47].

Established strategies for reducing incidence of CMD, such as use of virus-free cuttings and conventional breeding for genetic improvement of cassava using resistant landraces [17, 36, 46, 69, 71], have not been effective in reducing cassava yield loss. Genetic modification of cassava for introduction of resistance traits has been hampered by this non-model plant’s recalcitrance to transformation [49, 99]. Furthermore, these approaches are time-consuming, causing attention to shift to the use of newer biotechnological approaches such as CRISPR-mediated gene editing [77].

Certain cassava genotypes exhibit natural resistance or tolerance to cassava mosaic begomoviruses (CMBs), for example, tropical *M. esculenta *3 (TME3) [2] and Tropical Manihot Series (TMS) 96/0023 [30]), whereas others (cv.60444, T200, TMS 8017) are susceptible to CMBs and do not recover from infection (reviewed in [28, 48]. Although it is not clear which particular genes are involved in cassava’s response to SACMV infection, the availability of naturally-resistant cassava genotypes, transcriptomic data from infected susceptible and tolerant cassava genotypes [5], genome-wide association study data [94] and the cassava genome [14, 41, 75] enables the selection of putative host interacting genes for screening and testing.

Existing methods of cassava transformation are laborious, taking at least fifteen weeks from commencement of plant cultures to gene expression assaying [49, 99]. Transforming plant protoplasts instead of whole plants has a relatively shorter turnaround time, and is desirable because protoplasts show comparable cell-independent responses as whole plants [85, 98]. Transient transformation of plant protoplasts for rapid gene characterisation is well-established for several plant species [1, 38, 56, 68, 76, 96]. More recently, cassava protoplasts were used for rapid gene characterisation [96] because they are a demonstrably reliable system for correlating *in planta* activities. Further, co-transformation of plant protoplasts with multiple plasmid constructs is routinely conducted [18, 55, 91] and specifically plant protoplast co-transformation with virus infectious clones and plasmid constructs has been used to explore gene function during viral infection [20, 89].

We propose that simultaneous targeted mutagenesis and viral infection can facilitate characterisation of the genetic architecture during the diseased state, and if coupled in protoplasts, can provide a high-throughput rapid screening platform for genes that may be central to *in planta* host-virus interactions. Potential gene candidates can then be further validated *in planta* using virus-induced gene silencing (VIGS). To explore this, we targeted a cassava ubiquitin E3 ligase gene, *MeE3L* (*Manes.12g069400*), for CRISPR-mediated mutagenesis in SACMV-infected cassava protoplasts from the model cultivar (60,444), and susceptible (T200) and tolerant (TME3) African cassava landraces. *MeE3L* was targeted because ubiquitin RING E3 ligases play a central role in the hijacking and redirection of ubiquitination by geminiviruses [4, 31, 58, 90]. Moreover, *MeE3L* has previously been implicated in the response to stress [50, 73] and associated with the CMD2 resistance locus [57, 94]. We analysed primary *MeE3L* gene structure, predicted MeE3L protein tertiary structure, *MeE3L* expression and SACMV DNA accumulation to determine whether *MeE3L* may be involved in the response of cassava protoplasts to SACMV infection. Findings in this study demonstrated the suitability of the cassava protoplast system for high-throughput screening of the genes involved in cassava’s response to SACMV.

## Methods and materials

### CRISPR-Cas9 vector construction

Two genomic gRNA targets, gRNA1 (forward strand: GCGCAGATTCAAGCACTCGA) and gRNA2 (reverse strand: ACGTCCATTGGCGATGATAG),were identified using the CRISPOR version 4.7 web-based program (www.crispor.tefor.net; [29]) and used in designing a duplex sgRNA that included an *Arabidopsis thaliana* U6-26 promoter, the sgRNA scaffold and terminator for each gRNA sequence. Synthesis of the duplex sgRNA was outsourced to Inqaba Biotec (Pretoria, South Africa) and the duplex was cloned into the pCambia1380 vector. A *Cas9* insert (*Tobacco mosaic virus *promoter + *Cas9* gene + *eGFP* gene + *Hsp* terminator) from the pl1m-f2-p35s-cas9-egfp-nucleo-thsp Golden Gate vector was cloned into the pCambia1380-gRNA construct. The construct was confirmed by restriction digestion and sequencing.

### Protoplast isolation

Sterile nodal cultures of cassava (cv.60444, T200 and TME3 genotypes) were grown for 4 weeks at 28 °C (3,000 lx; 12/12 h light/darkness) on ½ Murashige and Skoog (MS) medium (2.2 g Murashige and Skoog Basal Medium, 2% sucrose, 0.002 mM CuSO_4_, 0.78% plant tissue culture agar) [67] to provide 3 independent biological replicates of each genotype. For each treatment, 0.3 g of fully expanded leaves were transversely sliced into 2–3 mm strips, which were plasmolysed by immersion in CPW9M medium (0.5 M mannitol, 27.2 mg KH_2_PO_4_, 100 mg KNO_3_, 150 mg CaCl_2_, 250 mg MgSO_4_, 2.5 mg Fe_2_(SO_4_)_3_.6H_2_0, 0.6 mg KI, 0.00025 mg CuSO_4_ per litre; pH 5.8) for 1 h [8]. The strips were vacuum-infiltrated in enzyme digestion solution (5 mM morpholinoethanesulphonic acid (MES), 1.6% cellulase, 0.8% macerozyme, CPW9M medium) for 30 min and then incubated at 25 °C in the dark at 40 rpm for 16 h. Protoplasts were released by shaking the digested tissue at 80 rpm for 5 min and purified by filtering through a 75 μm sieve. The filtrate was centrifuged at 100 *g* and the protoplast pellet washed twice in CPW9M medium. Protoplast integrity was checked using the Olympus BX 63 OM/FM microscope (Olympus Scientific Solutions, Massachusetts, USA), viability was determined by Evans’ Blue Dye staining [33], and quantification conducted via flow cytometry using the BD Accuri™ C6 flow cytometer (BD Biosciences, New Jersey, USA). Flow cytometric data were analysed using FCS Express 7 Research Edition software (Treestar, Inc, Oregon, USA). Protoplasts were resuspended in MMg solution (0.4 M mannitol, 15 mM MgCl_2_, 4 mM MES; pH 5.8) [96]) to a concentration of 10^4^ cells per ml.

### Protoplast transfection

For each of the 3 biological replicates for each cassava genotype, 15 μg of the CRISPR construct and 4 μg each of pBIN19-SACMV-DNA-A and pBIN19-SACMV-DNA-B infectious clones [12] were mixed with 1 mL of protoplasts and 25% polyethylene glycol 4000 (PEG4000), and incubated at room temperature for 20 min. The mixture was gently diluted with 3 volumes of W5 solution (154 mM NaCl, 125 mM CaCl_2_, 5 mM KCl, 2 mM MES, pH 5.8) and centrifuged twice at 100 *g* for 2 min. Protoplasts were resuspended in 300 μL of WI solution (4 mM MES, 0.5 M mannitol, 20 mM KCl, pH 5.8) [98] and incubated overnight in the dark at room temperature to induce gene expression. The expression of eGFP was checked using fluorescence microscopy to confirm protoplast transformation. Protoplasts were washed with CPW9M medium at 24 h post-transfection.

### Mutagenesis, viral load and gene expression assays

DNA was extracted from the transformed protoplasts 24 h post-transfection (hpt) using QIAzol Lysis Reagent according to a user-developed protocol (www.qiagen.com/it/resources/; Qiagen, Maryland, USA). Quantitative PCR (qPCR) for SACMV relative viral load quantitation using *Dpn*I-digested (ThermoFisher Scientific, Massachusetts, USA) DNA as template was performed in triplicate using forward (5` GGCTAGTTCCCGGATTACAT 3`) and reverse (5` GACAAGGACGGAGACACC 3`) primers, and 18S rRNA as the reference gene. The exonic region of *MeE3L* was amplified using the Phusion U Green Hot Start DNA Polymerase (ThermoFisher Scientific) with forward (5` CGCGCAGATTCAAGC 3`) and reverse (5` TGTCCACATGGAATGAAAG 3`) primers. Sequencing of amplicons on the ABI 3500XL Genetic Analyzer was outsourced to Inqaba Biotec (Pretoria, South Africa), and sequencing results were analysed for variation and alignment using TIDE web tool (https://tide.deskgen.com/; [16]) and MAFTT version 7 (https://mafft.cbrc.jp/alignment/software/, [79]) before being employed as query terms for protein structure and binding prediction using the I-TASSER On-line Server (https://zhanglab.ccmb.med.umich.edu/I-TASSER/, [97]) and protein similarity search in the Protein Data Bank [10]. Analysed sequences were used to construct a phylogenetic tree using the MEGA X software based on the Maximum Likelihood method and Jones-Taylor-Thornton (JTT) matrix-based model [40]. Frequency of clones with altered sequence was obtained by expressing a number of amplicons from a polyclonal mix with sequence alteration as a ratio of 10 amplicons sequenced. Mutations were determined by aligning amplicon sequences with wild-type reference AM560-2 [14] and TME3 (RefSeq ID: RSFT01000007, GenBank assembly GCA_003957995.1 (unpublished data)) *MeE3L* homologs. Alignment was conducted on MEGA-X [40] using the CLUSTAL W algorithm for multiple sequence alignment [44].

RNA was extracted from the transformed protoplasts 24 hpt using QIAzol Lysis Reagent and according to the manufacturer’s (Qiagen, Maryland, USA) protocol. First strand cDNA synthesis using RNA as template was performed using the RevertAid H Minus First Strand cDNA Synthesis Kit (ThermoFisher Scientific, Massachusetts, USA). Reverse transcriptase qPCR for *MeE3L* relative expression quantitation was performed in triplicate with cDNA as template using the Maxima SYBR Green/ROX qPCR Master Mix (2X) (ThermoFisher Scientific, Massachusetts, USA) with forward (5` CGCGCAGATTCAAGC 3`) and reverse (5` TGTCCACATGGAATGAAAG 3`) primers according to the manufacturer’s protocol using 18S rRNA as the reference gene. The qPCR data was analysed by one-way ANOVA and Tukey’s HSD test (p < 0.05). Relative expression and relative viral load of *MeE3L* and SACMV, respectively, were determined using the 2^−ΔΔCT^ method [54].

## Results

### Isolation and transformation of cassava protoplasts

**Fig. 1:**
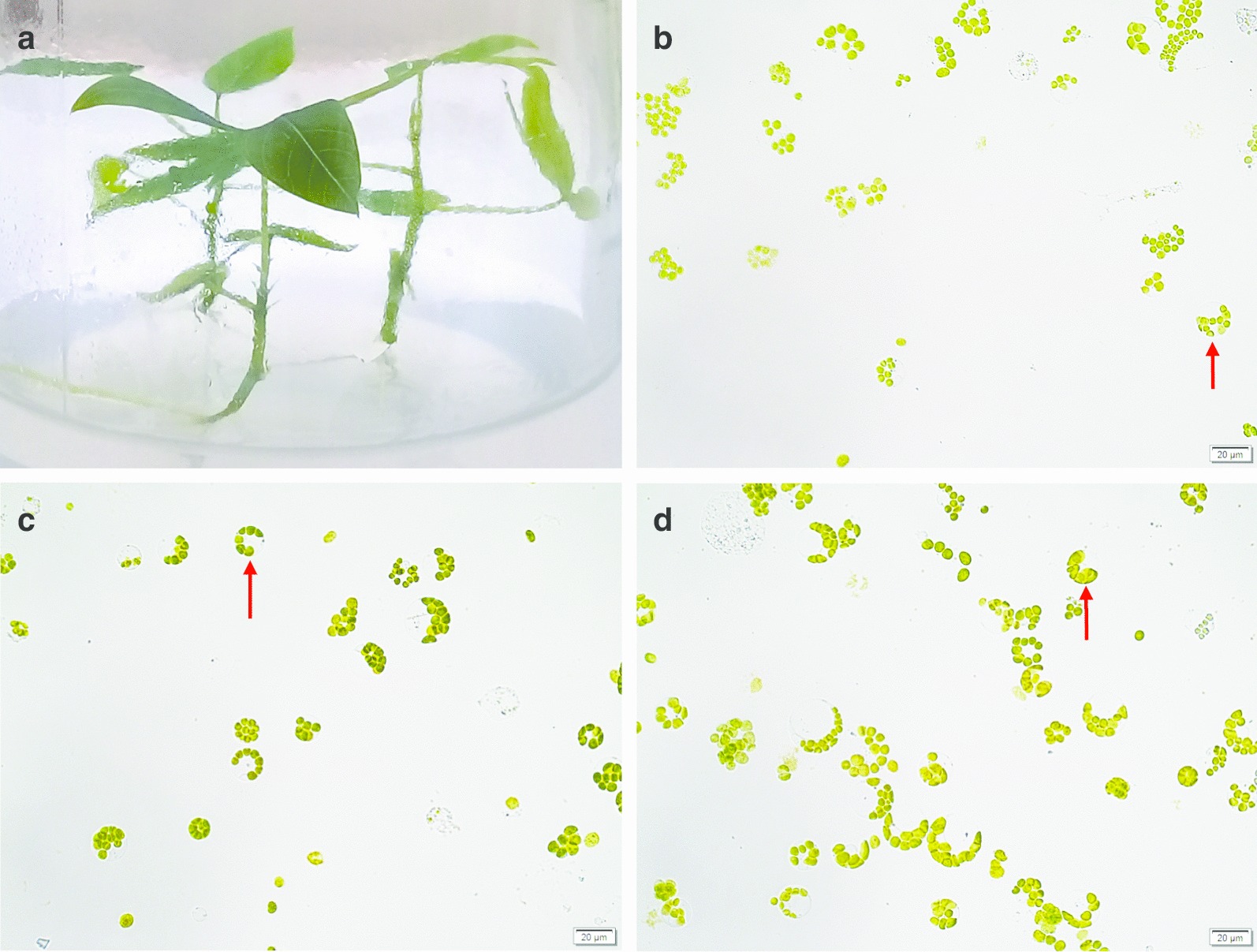
Cassava protoplast isolation from leaf mesophyll cells by 16 h-long enzymatic digestion. **a** *M. esculenta* 4-week old donor plants cultured on ½ Murashige and Skoog medium. **b** Protoplasts from model *M. esculenta *cv.60444 **c** Protoplasts from susceptible *M. esculenta *T200 **d** Protoplasts from tolerant *M. esculenta *TME3. Spherical protoplasts with chloroplasts around the edge of the cell membrane and central vacuole were observed (shown by red arrows). Protoplasts were visualised under bright field microscopy

**Fig. 2:**
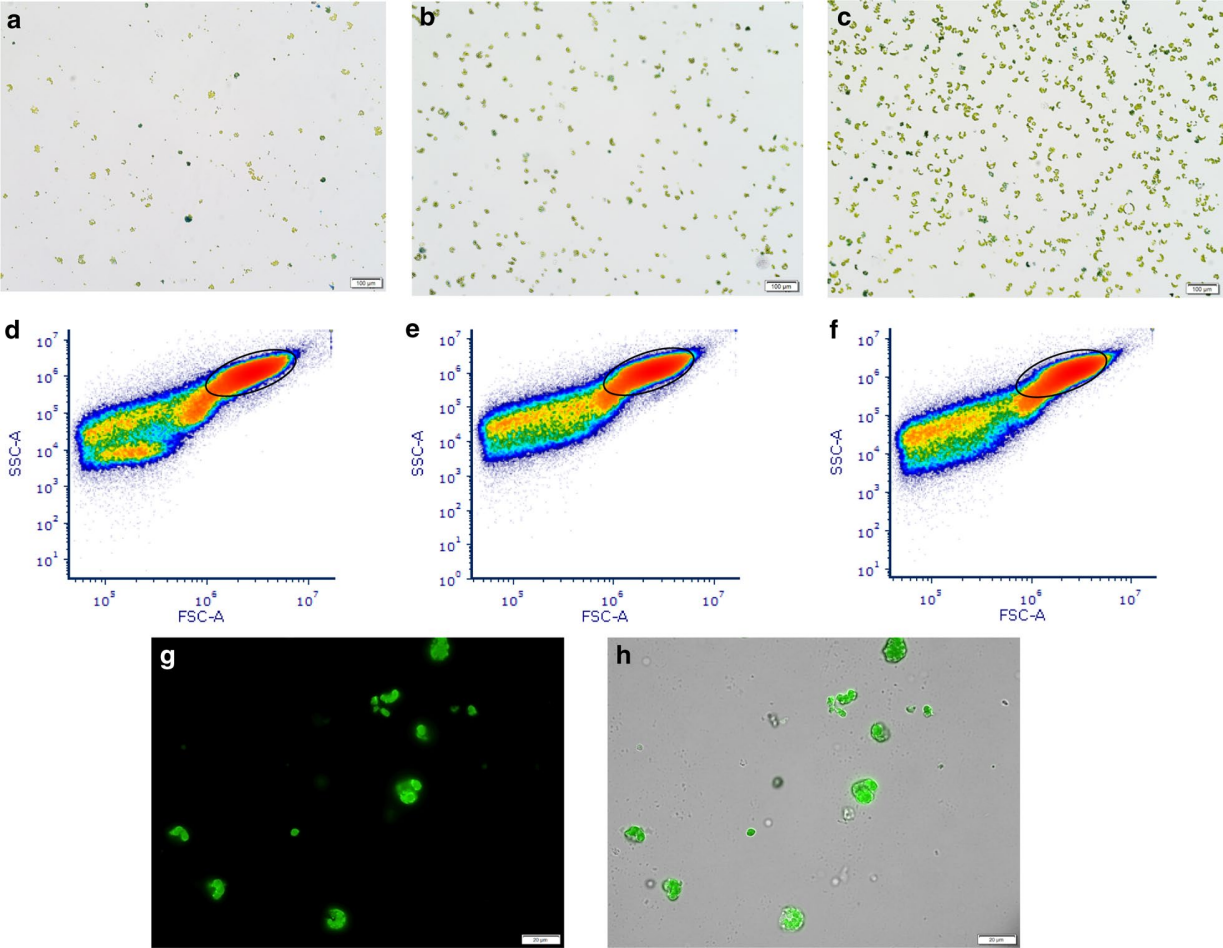
Analyses of viability, quality and transformation of cassava protoplasts. Viability of freshly isolated protoplasts was determined by Evans’ Blue Dye staining and visualisation under bright field microscopy. Analysis of protoplast quality was done by flow cytometric density measurement where events are discriminated by size and granularity, represented in log scale density plots. The size and shape of cassava protoplasts are measured by their effect on the forward scatter (FSC-A) and side scatter (SSC-A) of the laser. Stable transformation with the CRISPR construct was determined by fluorescence microscopy visualisation of eGFP fluorescence through the GFP filter and bright field. **a** Protoplasts from model *M. esculenta *cv.60444; **b** Protoplasts from susceptible *M. esculenta* T200 (**c**); Protoplasts from tolerant *M. esculenta* TME3. Non-viable cells are stained blue. **d** Plot of model *M. esculenta*c v.60444 protoplast density (**e**) Plot of susceptible *M. esculenta *T200 protoplast density (**f**) Plot of tolerant *M. esculenta *TME3 protoplast density. Circled regions correspond to desirable protoplasts. **g** *M. esculenta *T200 protoplasts visualised through the GFP filter (**h**) *M. esculenta* T200 protoplasts visualised through both the bright field and GFP filters

The *in vitro* growth of the cassava plantlets was conducted under controlled, sterile conditions and only young, expanded leaves were used as donors (Fig. 1a). Different sizes (~ 15–35 µm) of cassava protoplasts of round and irregular shape were observed (Fig. 1b–d). The majority of protoplasts were round with chloroplasts positioned around the perimeter of a central vacuole. The viability of protoplasts was at least 85% as shown by staining with Evans’ Blue Dye (Fig. 2a–c). Protoplast yields were 4.90–6.36 × 10^6^/g fresh weight (FW) (Additional File 1). The integrity of isolated protoplasts was analysed by flow cytometry forward versus side scatter (FSC vs SSC) gating [32] prior to transfection (Fig. 2d–f). The presence of protoplasts in the gated area as well as irregularly-shaped debris outside the gated area was detected. Approximately 10^4^ protoplasts of each cassava genotype were transfected with 15 µg eGFP-tagged CRISPR construct and/or 4 µg SACMV infectious clones using PEG-mediated transformation. Stability of transient expression was verified by detection of eGFP expression 24 hpt, showing that at least 90% of protoplasts had been successfully transformed (Fig. 2g, h).

### Structure and phylogenetic analysis of MeE3L in protoplasts from model, susceptible and tolerant cassava genotypes

**Fig. 3:**
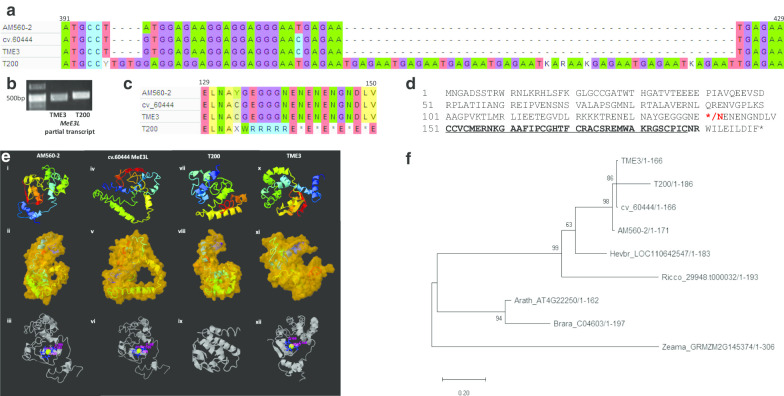
Primary structure, secondary structure and phylogenetic analysis of *MeE3L* and/or its protein product. Sequence alignment and agarose gel resolution of *MeE3L* partial gene and partial transcript respectively show a 53 bp insertion mutation in the lengthier susceptible T200 homolog that is absent in the reference AM560-2, model cv.60444 and tolerant TME3 homologs. Computational prediction of secondary, molecular and zinc-binding structures of MeE3L homologs shows significant differences between T200 structure and the other structures. N → C. Phylogenetic analysis shows significant evolutionary distance between susceptible T200 MeE3L and other plant MeE3L homologs. **a** Genomic nucleotide sequence alignment showing insertion mutation between nucleotides 397–398 and 422–423 in the susceptible T200 *MeE3L* homolog. **b** Agarose gel resolution of the PCR-amplified susceptible T200 and tolerant TME3 partial transcripts of *MeE3L* (**c**) Amino acid sequence alignment showing premature stop mutation at amino acid residue 141 in susceptible T200 MeE3L protein homolog (**d**) The reference AM560-2 MeE3L amino acid sequence. Asterisks denote stop codons in susceptible T200 (amino acid residue 141) and reference AM560-2 / model cv.60444 / tolerant TME3 (amino acid residue 200) homologs respectively. Red letters denote the first susceptible T200 MeE3L stop mutation at amino acid residue 141. Underlined letters denote the sequence adhering to the RING finger domain consensus sequence [CX_2_CX_(9–39)_CX_(1–3)_HX_(2–3)_CX_2_CX_(4–48)_CX_2_X]. (**ei**) Reference AM560-2 MeE3L homolog predicted secondary structure (**eii**) Reference AM560-2 MeE3L homolog predicted tertiary molecular structure (**eiii**) Zinc binding in RING domain of reference AM560-2 MeE3L (**eiv**) Model cv.60444 MeE3L homolog predicted secondary structure (**ev**) Model cv.60444 MeE3L predicted tertiary molecular structure (**evi**) Zinc binding in RING domain of model cv.60444 MeE3L (**evii**) Susceptible T200 MeE3L homolog predicted secondary structure (**eviii**) Susceptible T200 MeE3L predicted tertiary molecular structure (**eix**) Predicted ligand binding structure of susceptible T200 MeE3L (**ex**) Tolerant TME3 MeE3L homolog predicted secondary structure (**exi**) Tolerant TME3 MeE3L predicted tertiary molecular structure (**exii**) Zinc binding in RING domain of tolerant TME3 MeE3L [Predictions were run on the I-TASSER On-line Server (https://zhanglab.ccmb.med.umich.edu/I-TASSER/; [97]] (**f**) Evolutionary analysis of plant MeE3L homologs using Maximum Likelihood method and Jones-Taylor-Thornton (JTT) matrix-based model in MEGA X [40]. Bootstrap support was calculated from 1000 replicates. The tree is drawn to scale, with branch lengths measured in the number of substitutions per site

PCR amplification of the *MeE3L* partial transcript from leaves revealed that the susceptible T200 landrace homolog nucleotide sequence is slightly longer than the tolerant TME3 homolog (Fig. 3b). Sequencing of the exonic region of susceptible T200 *MeE3L* showed that this is due to a 53 bp insertion mutation of 9 TGAGAA nucleotide repeats that are absent in model cv.60444 and tolerant TME3 (Fig. 3a). The resulting frameshift introduces a stop codon corresponding to amino acid 141 (Fig. 3c, d). Computational analysis revealed that the truncated susceptible T200 MeE3L homolog is structurally distinct from the reference genome (AM560-2), model cv.60444 and tolerant TME3 MeE3L homologs (Fig. 3e). It is also not significantly similar in structure to any protein in the Protein Data Bank [10], and it is not confidently predicted to bind any ligands. The other three (AM560-2, cv.60444 and TME3) homologs are structurally closest to the cIAP1 inhibitor of apoptosis protein which contains a RING domain with E3 ligase activity for autoubiquitination and modulates cell death [25]. An analysis of the phylogeny of E3 ligase homologs in plants (Fig. 3f) reveals that although the MeE3Ls share *Hevea brasiliensis* as a common ancestor, the susceptible T200 homolog is significantly more evolutionarily distant from AM560-2 than the TME3 and cv.60444 homologs.

### Relative accumulation of SACMV DNA and relative *MeE3L* expression in transformed cassava protoplasts

**Fig. 4:**
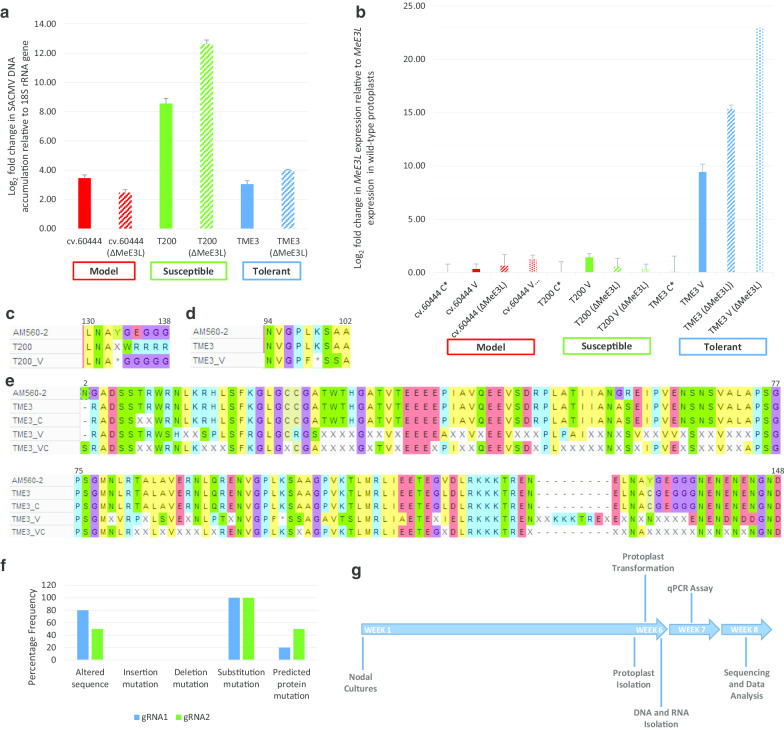
Assessment of viral DNA accumulation, relative *MeE3L* expression, and predicted MeE3L primary structure in transformed cassava protoplasts. **a** Relative DNA accumulation of SACMV- and SACMV + CRISPR-Cas9-transformed cassava protoplasts under different transformation conditions. Δ*MeE3L* = mutant CRISPR-edited *MeE3L*. Real-time qPCR was performed in triplicate using *Dpn*I*-*treated total DNA extracted from cassava protoplasts 24 hpt as template. **b** *MeE3L* relative expression levels in transformed cassava protoplasts. V = SACMV-transformed. Δ*MeE3L* = gene-edited *MeE3L*. C* = transformed with CRISPR construct lackingt gRNA duplex. RT-qPCR was performed in triplicate using total mRNA as template. **c** Stop mutation induced in SACMV-infected susceptible T200 *MeE3L*. **d** Stop mutation induced in SACMV-infected tolerant TME3 *MeE3L*. **e** The predicted amino acid sequence of tolerant TME3 MeE3L at reference sequence positions 2–148 showing multiple mutations in SACMV-infected variant. V = SACMV-infected. C = gene-edited. Sequence alignment was conducted in MEGA-X [40]. **f** Frequency and types of mutation at target gRNA sites from CRISPR-transformed protoplasts. Frequency of clones with altered sequence was obtained by expressing number of amplicons from a polyclonal mix with sequence alteration as a ratio of total amplicons (n = 10 per genotype) sequenced. Mutations were determined by aligning amplicon sequences with wild-type reference AM560-2 [14] and TME3 (RefSeq ID: RSFT01000007,GenBankassembly GCA_003957995.1 (unpublished data)) *MeE3L* homologs. Alignment was conducted on MEGA-X [40] using the CLUSTAL W algorithm for multiple sequence alignment [44]. **g** Timeline for rapid screening of genes associated with the response to *South African cassava mosaic virus* in cassava

Tukey’s HSD test showed significant differences in the qPCR data derived from SACMV-, CRISPR- and SACMV + CRISPR-transformed protoplasts (p < 0.05) (Additional File 2). Relative SACMV DNA accumulation in wild-type protoplasts was highest in susceptible T200 at eightfold, compared to threefold relative to the 18S rRNA gene in model cv.60444 and tolerant TME3 at 24 hpt. There was a substantial increase in accumulation of SACMV DNA in model cv.60444, susceptible T200 and tolerant TME3 (12-fold and fourfold respectively) in CRISPR-transformed protoplasts (Fig. 4a). *MeE3L* expression was significantly upregulated with a log fold change of 1 and 9 in susceptible T200 and tolerant TME3 SACMV-infected protoplasts relative to *MeE3L* expression in untransformed protoplasts, respectively. There was significant upregulated expression of *MeE3L* in tolerant TME3 but no significant effect on susceptible T200 *MeE3L* expression in CRISPR-transformed protoplasts. No significant change in *MeE3L* expression was observed under all conditions in model cv.60444 (Fig. 4b).

### Predicted MeE3L primary structure in transformed cassava protoplasts

Predicted MeE3L protein primary structures based on the *MeE3L* genomic sequence in SACMV-infected protoplasts showed stop mutations (amino acid residues 133 and 99 respectively) (Fig. 4c, d) upstream of the RING domain in susceptible T200 and tolerant TME3 variants, compared to uninfected controls and reference sequence AM560-2. Sequencing of MeE3L from SACMV-infected tolerant TME3 protoplasts revealed multiple random single base mutations along the length of MeE3L which translate to substitutions by altered amino acids (Fig. 4e). CRISPR editing efficiency was determined based on Sanger sequencing of 10 amplicons from a polyclonal mix. Sequencing indicated 80% and 50% mutation frequency for the gRNA1 and gRNA2 targets respectively. All mutations were substitution mutations and translated to 20% and 50% mutation efficiency for the gRNA1 and gRNA2 targets respectively in the predicted protein sequence (Fig. 4f). The turnaround time from commencement of plant tissue culture to gene expression assaying was 7 weeks (Fig. 4g).

## Discussion

### African cassava landraces susceptible and tolerant to SACMV are amenable to enzymatic protoplast isolation and PEG-mediated transformation

Protoplasts were chosen for this transient gene expression study because they can conveniently and efficiently be transformed with several DNA constructs simultaneously, and they allow higher resolution imaging compared to cells in intact tissue [27]. Additionally, they can be used for high-throughput efficient screening of candidate genes, and those genes that show an effect can then be silenced by virus induced gene silencing (VIGS) *in planta*, which takes considerably longer (3–4 months) and requires more complicated procedures for the non-model host cassava. Leaf mesophyll was used as the source of protoplasts because SACMV exerts its effects mainly in the leaves, where symptoms arise. Leaf mesophyll protoplasts therefore provide functional information [27] relating to the effect of SACMV at leaf tissue level. Round and irregularly-shaped protoplasts of different sizes were observed (Fig. 1b–d), although spherical leaf mesophyll protoplasts dominated, as generally reported [96].

A previously determined enzyme concentration (1.6% cellulase, 0.8% macerozyme) that is suitable for obtaining the optimum number of viable protoplasts was used for leaf cell wall digestion [96]. The viability of protoplasts in this study was at least 85% although cassava protoplast viability of up to 95% has been reported (International Plant Research Institute, 1984). The long digestion period (16 h) was ruled out as the cause of death for ~ 15% of protoplasts as this reportedly does not induce serious damage in protoplasts [87]. It has been reported that micro-propagated plants grown* in vitro* lack epicuticular wax and thus allow rapid enzyme penetration [40]. Cassava, however, has a thick epicuticular layer which necessitates the long digestion period of 16 h compared to 0.3-1 h for Arabidopsis [95]. Macerating enzymes such as macerase are known to cause wound reactions in protoplasts because of their degradation of the cell wall, which may lead to necrosis [34], while cellulase is known to exert inadequate enzymatic activity at low concentrations and higher concentrations have no benefit or detriment [88]. Therefore, a balance between digestion enzyme concentration and viability is essential in order to obtain the optimum yield of viable good quality protoplasts. Cassava protoplast viability presented herein may differ from previously reported percentages possibly due to differences in the cassava genotypes used, and in this particular case could be due to the particular physiological characteristics of the African cassava landraces from which protoplasts were derived.

Although lower than the previously reported yields of 4.4 × 10^7^ protoplasts/g FW leaves from *M. esculenta *cv. South China 8 [96] and 1.9 × 10^7^ protoplasts/g FW leaves from *M. esculenta* cv. M. Thai 8 [8], the protoplast yields in this present study (4.90–6.36 × 10^6^/g FW) were sufficient to provide the recommended number of protoplasts (10^4^–10^7^) required for each transfection [98]. Both pre-treatment of leaves in the dark (24–72 h) [84] and vacuum infiltration before enzyme digestion [68] have been shown to intensify enzyme penetration in bean leaves. We found that pre-treatment resulted in release of much undesirable plant debris alongside protoplasts, and that vacuum infiltration did indeed help increase protoplast yield. Flow cytometry indicated a high concentration of protoplasts compared to irregularly-shaped debris. Based on microscopy images of purified protoplasts (Fig. 1b–d), the irregular debris outside the flow cytometry gated area was deemed to be free chloroplasts, plasmolysed cells, undigested cell wall fragments and other aggregates arising from the long digestion period of leaf material.

Stability of protoplast transformation was confirmed by expression of eGFP from the CRISPR construct at 24 hpt following PEG-mediated transfection of about 10^4^ protoplasts with 15 µg CRISPR construct and 4 µg SACMV infectious clones. Protoplasts were deemed unsuitable for further analysis from 36 hpt as they rapidly lost viability. PEG-mediated plant protoplast transfection with plasmid DNA is a well-established procedure (Hayashimoto *et al*. 1990; [55]) and a popular protocol uses 10 μg DNA to transfect 2 × 10^4^ Arabidopsis protoplasts [98]. At least 5 µg of plasmid DNA have previously been used to transform 10^6^ tobacco protoplasts with *African cassava mosaic virus* (ACMV) (Ermak *et al*. 1993) and *Cowpea mosaic comovirus* [93]. Highly efficient co-expression of multiple constructs in plant protoplasts has been reported [18, 91] and virus infectious clones have also been used in conjunction with other plasmid constructs for co-inoculation of plant protoplasts [20]. *Nicotiana tabacum* protoplasts have been co-transformed with 5 µg eGFP construct, 3 μg siRNA and 4 μg each of ACMV or East African cassava mosaic virus (EACMV) with DNA/RNA extraction at 36 and 48 hpt [89]. To our knowledge, the present study is the first report of co-transformation of cassava protoplasts with a CRISPR construct and geminivirus infectious clones. The number of constructs used for transformation can be increased for high-throughput studies to target multiple genes simultaneously since it has been shown that transformation efficiency is independent of plasmid amount [55].

The percentage mutation frequency of 50–80% at targeted sites in the cassava genome is comparable to the 70% and 60% attained in cassava [96] and *N. tabacum *[52] protoplasts respectively, and considerably less than the 100% previously obtained in cassava plants [70]. CRISPR targets with GC content greater than 50% are known to achieve higher efficiency than those with less than 50% [59], and this may explain why gRNA1 target sequence (55% GC content) has considerably higher mutation frequency than gRNA2 target sequence (50% GC content).

### The *M. esculenta* T200 *MeE3L* encodes a truncated RING-less protein due to a nonsense mutation

The frameshift resulting from the insertion mutation in susceptible T200 *MeE3L* introduces a stop codon upstream of the RING domain, thus encoding a truncated protein in which the C3HC4-type RING finger motif is absent. Essentially, the E3 ligase domain in the T200 *MeE3L* would not be translated because of this mutation, making its potential protein product non-functional with respect to this E3 ligase activity. We were able to amplify the T200 *MeE3L* exon from cDNA, showing that this gene is transcribed, but we have no evidence of its translation or lack thereof. It is proposed that the loss of this substantial portion of the T200 MeE3L C-terminal region would not only avert E3 ligase activity but may also alter the spatial chemical conformation necessary for any other interactions (such as binding the ubiquitin-conjugated E2 and substrate) to occur. The protocol in this present study enables gene sequence comparison among wild type cassava genotypes, to determine the presence of single nucleotide polymorphisms (SNPs) or other forms of mutations, and to form a basis for *in planta* exploration of phenotypic differences among cassava genotypes.

The AM560-2, TME3, and cv.60444 cassava genotypes have previously been shown to cluster together under their nearest ancestor, *Hevea brasiliensis *[14]. The evolutionary history of T200, a southern African landrace, is unknown and phylogenetic analysis suggests that the T200 *MeE3L* evolved after the cv.60444 and TME3 variants. It is known that wild plants in natural ecosystems co-evolve with their virus partners. While it is recognized that there is a relationship between virus virulence/pathogenicity and co-adaptation to plant hosts (Sacristan and Garcia-Arenal 2008), information regarding how viruses apply selective pressure to alter plant susceptibility is not known. A study of *Drosophila* and its host-specific viruses found that coevolution may cause sustained genetic variation in susceptibility [26]. This may explain why a southern African cassava landrace is highly susceptible to SACMV that appears to have migrated south from its origin, suspected to be in east Africa or the south-west Indian Ocean islands such as Madagascar [24] that are geographically separated from the African continent [45]. South African cassava mosaic virus is a recombinant between *East African cassava mosaic virus* and two other unknown geminiviruses which contributed the AC4 and IR regions [12], and moved southwards into Mozambique, Zimbabwe and South Africa where it may have encountered the T200 landrace. Subsequent to its first discovery in South Africa, SACMV has been reported in Zimbabwe [15] and Madagascar [24]. It is known that infection with a new recombinant begomovirus requires the host to adjust to minor or major differences in virus-host interactions [64]. It is suggested that the T200 landrace and SACMV may still be in the process of co-adaption, which would explain why T200 exhibits extreme susceptibility to SACMV. We speculate that the *MeE3L* is either a paralog in T200 or it was introgressed from a wild relative in southern Africa.

### SACMV DNA accumulation in cassava protoplasts is genotype-dependent

Quantitative PCR is a well-established method for precise quantitation of viral DNA amount in infected tissue and it requires a host reference gene with stable expression patterns under experimental conditions as the internal control for correct data normalisation [65]. Data from the qPCR measurement of SACMV DNA accumulation (relative to the 18S rRNA gene) show that SACMV DNA accumulates in cassava protoplasts, correlating well with previous reports of geminivirus DNA accumulation *in planta* and* in vitro*. Quantitative detection of *African cassava mosaic virus* and *East African cassava mosaic virus* using qPCR has been reported [72] and SACMV titre, in particular, has been assayed *in planta* in Arabidopsis [74] and cassava [5]. Replication of the geminivirus, *Cassava brown streak virus,* in cassava leaf mesophyll protoplasts has been assayed at 6 hpt [7] and it has been reported that there was significant viral DNA accumulation in tobacco BY-2 protoplasts 36 and 48 hpt by co-inoculating with infectious ACMV and EACMV clones and siRNA [89]. The present study is the first to report accumulation of SACMV DNA in cassava protoplasts.

Based on previously reported *in planta* evidence [5] and the known presence of a CMD2 locus in tolerant TME3 [2], it was expected that SACMV DNA accumulation would be genotype-dependent and significantly lower in TME3 than in the model cv.60444 and susceptible T200 protoplasts. Interestingly, there was differential SACMV accumulation in CRISPR-transformed cassava protoplasts expressing the gene-edited *MeE3L*. The upregulation of SACMV DNA accumulation in susceptible T200 and tolerant TME3 in the presence of mutant *MeE3L* suggests a role for *MeE3L* as one of the host genes involved in the response to SACMV infection. CRISPR-associated modification of *MeE3L* may enhance SACMV DNA accumulation in susceptible T200 and tolerant TME3 by interfering with the ubiquitin proteasome system-dependent tolerance/resistance response mechanisms of cassava.

### Viral activity and gene editing of *MeE3L* affect the expression of *MeE3L*

Geminiviruses elude plant defense mechanisms by hijacking and redirecting ubiquitination, and interfering with responses regulated by ubiquitin E3 ligases (including responses to jasmonates, auxins, gibberellins, ethylene, abscisic acid) [58]. It follows then that alterations to E3 ligase genomic sequences may alter E3 ligase expression patterns during viral infection, as viruses are known to modulate RNA levels to enhance infection [90]. Both plant viruses and CRISPR systems are known to induce mutations in the genome [22, 60, 82], and the employment of both against the *MeE3L* would provide an indication whether *MeE3L* may be involved in the plant’s response to SACMV.

Previously, plant E3 ligases have been shown to be induced by viral infection [19, 23, 43] and plant defence elicitors [51, 80]. It is known that geminiviruses interact with plant E3 ligases and induce their up- or down-regulation to promote infection or undergo degradation [43, 58, 86]. Results presented herein indicate that in TME3 protoplasts, *MeE3L* expression is upregulated during SACMV infection. The concurrent CRISPR-mediated gene editing of *MeE3L* and infection with SACMV appears to induce increased expression of the *MeE3L*, suggesting that *MeE3L*’s specific base sequence is important for the interaction between the virus and the plant host. The muted response of the T200 *MeE3L* to all treatments was expected given its nonsense mutation which silences the RING domain responsible for E3 ligase activity. However, the muted response of model cv.60444 *MeE3L* was unexpected and suggests that this *MeE3L* sequence variant is not responsive to SACMV infection.

The *MeE3L* homolog sequences in SACMV-infected protoplasts reflect a silenced RING domain, suggesting that SACMV may possibly induce silencing of the RING domain in order to achieve full infection of the host. The concomitant increase in SACMV DNA accumulation and gene-edited *MeE3L* in TME3 points to the response of *MeE3L* to SACMV being more directed at advancing susceptibility. There is evidence for geminiviral (*Tomato yellow leaf curl sardinia virus*) silencing of a plant E3 ligase, RHF2A, to promote infection [58] and impairment of plant defence during *Cabbage leaf curl virus* (CaLCuV) infection due to inhibition of a RING E3 ligase [81]. The present study provides further evidence that geminiviruses may interfere with activity of plant E3 ligases.

### SACMV’s interaction with a tolerant cassava genotype induces numerous mutations in *MeE3L*

Functions of E3 ligases in regulating immunity systems are orchestrated at the interface of host-virus interactions [100] and some of these interactions occur in the nucleus [42]. Sequencing of genomic *MeE3L* from SACMV-infected TME3 protoplasts revealed multiple random single base mutations along the length of *MeE3L*, which translate to amino acid substitution (Fig. 4e). While these mutations do not alter the reading frame, they are predicted to silence the whole protein and not just the RING domain. The resulting disordered protein would presumably not only lack RING E3 ligase activity, but also the E2 and substrate binding activity. These mutations were present and similar in all 10 genomic DNA amplicons derived from the polyclonal mixes of each of 3 biological replicates. Similar mutations encoding multiple stop codons have been observed in an Argonaute 4-encoding gene (*Manes.18g121900*) from SACMV-transformed tolerant TME3 protoplasts (unpublished data; Chatukuta and Rey), indicating that other host genes may be similarly affected by SACMV infection.

Interestingly, the discovery of mutations in genomic DNA presented herein possibly point to a yet unknown geminivirus-induced host mechanism for genome editing. Geminiviruses are known to induce the expression of genes related to repair of double-stranded breaks (DSBs) and DNA synthesis [57], and to promote somatic homologous recombination [78]. Some E3 ligases and viral proteins can localise to the nucleus, such as the tobacco E3 ligase, NtHUB1 which has a nuclear localisation sequence, is recruited by geminiviral Rep protein, and co-localises and interacts with the Rep protein to monoubiquitinate cellular chromatin and thus enable infection [42]. The viral coat protein, CP, also has a nuclear localisation signal, can localise in the nucleolus and nucleoplasm, and facilitates entry of ssDNA into the nucleus [39, 92]. However, the mechanisms for SACMV-mediated gene mutation induction in cassava protoplasts are yet to be investigated.

### The response of *MeE3L* to SACMV is virus- and host-specific

Ubiquitin ligases are abundant in plants and provide substrate specificity to target particular proteins. In Arabidopsis alone, RING E3 ligases make up 499 out of over 1,500 E3 ligases [62]. A comparison of E3 ligase and E3 ligase complex-associated gene expression during other plant geminivirus infection studies (Additional File 3) was conducted to determine whether *MeE3L*’s response to SACMV is geminivirus-specific or host-dependent.

In susceptible cassava, E3 ligase expression is downregulated during SACMV infection at early, middle and late time points (12, 32 and 64 days post infection (dpi)) but there is no differential expression of E3 ligases in tolerant cassava at any time point [5]. However, no differential expression of E3 ligases is recorded during SACMV infection of Arabidopsis which is susceptible (Pierce and Rey 2013). A study of transcriptomic responses to geminivirus *Tomato leaf curl New Delhi virus* (ToLCNDV) infection in potato found that five E3 ligases in the susceptible cultivar and two in the tolerant cultivar are upregulated at 30 dpi [35] while the geminivirus *Tomato yellow leaf curl virus* (TYLCSV) has been shown to induce upregulation of E3 ligases in susceptible tomato at 42 dpi, except in the case of a *CUL1* which is downregulated [63]. A transcriptome study of Arabidopsis during geminivirus CaLCuV infection found that, out of 1570 E3 ligases, 149 were up-regulated and 23 were downregulated [9]. The CaLCuV AC2 protein, in particular, induces downregulation of two E3 ligases in Arabidopsis [53]. These findings, together with the current study, prove that plant E3 ligase responses to geminivirus infection are neither uniform nor similar, but they vary according to the specific geminivirus and host involved in the interaction.

Responses of cassava to the ssRNA potyviruses *Cassava brown streak virus* (CBSV) and *Ugandan cassava brown streak virus* (UCBSV) with respect to E3 ligase expression variably show both downregulation and upregulation in the susceptible varieties. Interestingly, there is no differential expression of E3 ligases in resistant cassava varieties except in Kaleso where a *CUL1* is upregulated and a *RZPF34* is downregulated [6, 7, 61]. This variable expression of E3 ligases with respect to the virus in the same host suggests that while responses to viral infection are host-dependent, they are also modulated according to the particular virus infecting the plant.

SACMV infection *in planta* is associated with occurrence of severe symptoms leading to persistent severe infection in susceptible T200 and mild symptoms with recovery at 67 days post infection (dpi) in tolerant TME3 [5]. The SACMV-induced genetic mutations and differential expression of *MeE3L* post-infection in TME3 and T200 indicate that it is one of the genes involved in the plant’s response to the virus. *In planta* proteome data from our laboratory shows that during SACMV infection, an E3 ligase (*Manes.08G075100*) is upregulated in susceptible T200 and downregulated in tolerant TME3 cassava plants at 32 and 67 dpi (unpublished data; Rey), supporting indications from the protoplast system that E3 ligases are responsive to SACMV infection.

### Limitations

This protocol presented herein suffers some limitations due to the independent cell nature of protoplasts and the short-lived viability of cassava protoplasts in particular. It cannot be used to study cell wall-related genes, cell-to-cell signalling, intercellular movement, long-term responses, or long-term stability of CRISPR-induced gene edits. Further, the use of mesophyll protoplasts may not be suitable to correlate responses in other organs such as roots or flowers. The widely used T7 endonuclease I (T7EI) assay for detecting gene editing activity produced inconclusive results for this present study, and therefore gene editing was indicated by restriction digestion and confirmed by sequencing. It has been reported that CRISPR-Cas9 activity is more accurately reflected by Next Generation Sequencing (NGS) [83]. *In planta* validation of results from this protoplast system protocol, particularly overexpression and virus-induced gene silencing (VIGS) of targeted genes, as well as functional and interaction studies, must be conducted to confirm the specific roles played by candidate genes in the host-virus interaction.

## Conclusions

We have developed a simple and faster protocol for CRISPR-mediated transient gene expression assaying in cassava protoplasts infected with SACMV. While existing gene editing protocols for cassava plants take about 15 weeks, our protoplast-based method takes 7 weeks to provide experimental data that is suitable for screening candidate genes and informing *in planta* functional genomics studies. Using this protocol, we show that SACMV DNA accumulation in cassava protoplasts is genotype-dependent and it induces silencing of the *MeE3L* RING domain in susceptible T200 and tolerant TME3 landraces. We provide evidence for differential expression of native and mutant *MeE3L* during SACMV infection of cassava protoplasts. We also reveal that a SACMV-susceptible southern African cassava landrace (T200) expresses a mutant *MeE3L* with a silenced RING domain.

While this protocol cannot account for events relating to cell-to-cell signalling and movement, it does provide a basis for tentative identification of genes that respond to geminiviral infection in cassava. It may be adapted for high-throughput screening by targeting several genes simultaneously using a CRISPR multiplex approach.

## Supplementary information

**Additional file 1:** Protoplast yields, CRISPR-induced mutations and E3 ligase DE proteome data.

**Additional file 2:** ANOVA and Tukey's HSD analyses of qPCR data.

**Additional file 3:** E3 ligase and E3 ligase complex-associated gene expression during plant virus infection.

## Data Availability

The datasets used and/or analysed during the current study are available from the corresponding author on reasonable request.

## Abbreviations

AC4: Post-transcriptional gene silencing suppressor protein
ACMV: *African cassava mosaic virus*
APC: Anaphase-promoting complex
APC2: APC Regulator of WNT Signaling Pathway 2
ASK: Arabidopsis SKP1-related
BLAST: Basic local alignment search tool
bp: Base pair
BSCTV: *Beet severe curly top virus*
C2: Transcriptional activator protein
C4: Post-transcriptional gene silencing suppressor protein
CaLCuV: *Cabbage leaf curl virus*
CCNF: Cyclin F
cIAP1: Cellular inhibitor of apoptosis protein 1
CMD: Cassava mosaic disease
CPW9M: Cell and Protoplast Washing 9 Mannitol
CRISPR: Clustered regularly interspaced short palindromic repeats
CRL: Cullin ring
CUL1: Cullin 1
CUL4-DDB: Cullin4-damaged DNA-binding protein
cv: Cultivar
DBS: Double-stranded break
DDB1: DNA damage-binding protein 1
DNA: Deoxyribonucleic acid
dpi: Days post infection
DUB: Deubiquitinating enzyme
EACMV: *East African cassava mosaic virus*
eGFP: Enhanced Green Fluorescent Protein
FAO: Food and Agriculture Organisation
FW: Fresh weight
g: Gram
*g*: Standard acceleration due to gravity
GFP: Green Fluorescent Protein
h: Hour
HECT: Homologous to E6-associated protein C-terminus
hpt: Hours post-transfection
Hsp: Heat shock protein
IAP: Inhibitor of apoptosis protein
IR: Intergenic region
I-TASSER: Iterative Threading ASSEmbly Refinement
JTT: Jones-Taylor-Thornton
µg: Microgram
µM: Micromolar
M: Molar
MeE3L: *Manihot esculenta* E3 Ligase
MEGA X: Molecular Evolutionary Genetics Analysis X
mg: Milligram
min: Minute
ml: Millilitre
mM: Millimolar
MMg: Mannitol magnesium
N → C: Amino terminus to carboxy terminus
NbSKP1: *Nicotiana benthamiana* S-phase kinase-associated protein 1
NLR: Nucleotide-binding leucine-rich repeat
NMD: Nonsense-mediated mRNA decay
NtHUB1: *Nicotiana benthamiana* Homologous to ubiquitin protein 1
PCR: Polymerase chain reaction
PEG: Polyethylene glycol
qPCR: Quantitative polymerase chain reaction
QTL: Quantitative trait locus
R protein: Resistance protein
RBR: RING-between-RING RING
RBX1: RING-box protein 1
Rep: Replication-associated protein
RHF2A: RING-H2 finger E3 ubiquitin ligase protein
RING: Really Interesting New Gene
RNA: Ribonucleic acid
rpm: Revolutions per minute
rRNA: Ribosomal RNA
RZPF34: RING zinc finger protein 34
SACMV: *South African cassava mosaic virus*
SCF: Skp, Cullin, F-box containing complex
sgRNA: Single guide RNA
siRNA: Small interfering RNA
ssRNA: Single stranded RNA
T200: Tropical *Manihot esculenta* 200
TIDE: Tracking of indels by decomposition
TME3: Tropical *Manihot esculenta *3
ToLCNDV: *Tomato leaf curl New Delhi virus*
TYLCCV: *Tomato yellow leaf curl China virus*
TYLCSV: *Tomato yellow leaf curl Sardinia virus*
UCBSV: *Ugandan cassava brown streak virus*
UPS: Ubiquitin proteasome system
UTR: Untranslated region
βC1: Movement protein
