# Supplementary material for: A cassava protoplast system for screening genes associated with the response to South African cassava mosaic virus
Source: Virol J. 2020 Nov 23;17:184. doi: 10.1186/s12985-020-01453-4 (PMC7685591; doi:10.1186/s12985-020-01453-4)
Supplement: Supplementary file 1 — Additional file 1: Protoplast yields, CRISPR-induced mutations and E3 ligase DE proteome data. [file 12985_2020_1453_MOESM1_ESM.pdf]

# A Cassava Protoplast System for Screening Genes Associated with the Response to *South African Cassava Mosaic Virus*.

Patience Chatukuta, Marie Emma Chrissie Rey*
School of Molecular and Cell Biology, University of the Witwatersrand, South Africa
*Corresponding author

## ADDITIONAL FILE 1

1. Protoplast yields as determined by flow cytometric quantification

| | Plot 1 (FSC-A/SSC-A) | | | |
|---|---|---|---|---|
| | All | | | |
| | Count | Volume (µL) | Events / µL | % of This Plot |
| A02 cv.60444 untran... | 1,000,000 | 16 | 62500 | 100.00% |
| A03 T200 untransfor... | 1,000,000 | 18 | 55556 | 100.00% |
| A04 TME3 untransfor... | 1,000,000 | 18 | 55556 | 100.00% |

Additional Fig. 1: Enumeration of events in protoplast solutions (A02 = *Manihotesculenta* cv.60444; A03 = *M. esculenta* T200; A03 = *M. esculenta* TME3). Events were detected on the BD Accuri™ C6 Flow Cytometer (BD Biosciences, San Jose, CA, USA).

## 2. Mutations induced by CRISPR-mediated gene editing in *MeE3L*.

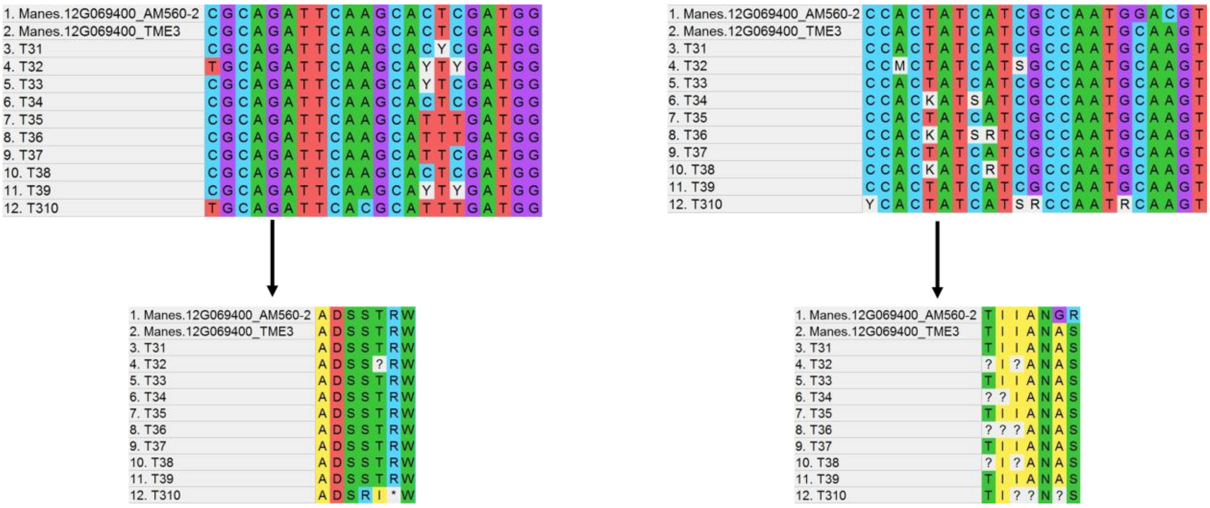


Additional Fig. 2: Alignment of 10 TME3 *MeE3L* genomic DNA sequences and their predicted amino acid sequences from transformed protoplast polyclonal mix against cassava reference genome AM560-2 (Bredeson *et al*., 2016) and cassava TME3 genome (RefSeq ID: RSFT01000007; GenBank assembly GCA_003957995.1 (unpublished data)) *MeE3L* homologs. Target sites for gRNA1 (forward strand: GCGCAGATTCAAGCACTCGA) and gRNA2 (reverse strand: ACGTCCATTGGCGATGATAG) are shown. Sanger sequencing was conducted on the ABI 3500XL Genetic Analyzer platform by Inqaba Biotec (Pretoria, South Africa). Alignment was conducted on MEGA-X (Kumar *et al*., 2018) using the CLUSTAL W algorithm for multiple sequence alignment (Larkin *et al*., 2007).

## 3. Differential expression of an E3 ligase protein *in planta* during SACMV infection

Summary of proteome study method: Cassava T200 and TME3 genotype plants were grown as described in Allie *et al*., 2014. Three independent SACMV infection trials were done and leaves sampled at 32 and 67 dpi. Samples were analysed in triplicate. Proteins were extracted using HEPES buffer and precipitated using the TCA/acetone method. Mass spectrometry analysis was conducted by the Centre for Proteomic and Genomic Research (CPGR, Cape Town, South Africa) using a label-free quantitative (LFQ) approach to identify fold changes in proteins.
